# A Commentary about Lessons Learned: Transitioning a Therapy Dog Program Online during the COVID-19 Pandemic

**DOI:** 10.3390/ani11030914

**Published:** 2021-03-23

**Authors:** Colleen Dell, Linzi Williamson, Holly McKenzie, Ben Carey, Maria Cruz, Maryellen Gibson, Alexandria Pavelich

**Affiliations:** Department of Sociology, University of Saskatchewan, Saskatoon, SK S7N 5A5, Canada; linzi.williamson@usask.ca (L.W.); holly.mckenzie@usask.ca (H.M.); ben.carey@usask.ca (B.C.); maria.cruz@usask.ca (M.C.); maryellen.gibson@usask.ca (M.G.); alexandria.pavelich@usask.ca (A.P.)

**Keywords:** virtual program, mental health, evaluation, social media, social support, isolation

## Abstract

**Simple Summary:**

The purpose of this commentary is to highlight the lessons our team learned through transitioning a university campus’ therapy dog program from being delivered in-person to a novel online format during the COVID-19 pandemic. By connecting therapy dog teams virtually with program participants, we aimed for participants to continue to experience feelings of love, comfort and support as occurred in in-person programming, and gain knowledge about the best evidence surrounding mental health care during a pandemic. Through a combined process-outcome evaluation and subsequent needs assessment of the online program, and reflecting on our collective experiences, we learned several lessons regarding program personnel needs, therapy dog handler training and support requirements, and online programming prerequisites. These learning experiences continue to inform our current delivery of the program online and are applicable to other programs considering the same.

**Abstract:**

In 2015, the University of Saskatchewan PAWS Your Stress Therapy Dog program partnered with St. John Ambulance for therapy dog teams to visit our campus and offer attendees love, comfort and support. We recognized at the start of the COVID-19 pandemic that students, staff and faculty may require mental health support, particularly with the challenges of isolation and loneliness. In response, our team transitioned from an in-person to a novel online format at the start of the COVID-19 pandemic. We designed online content for participants to (1) connect with therapy dogs and experience feelings of love, comfort and support as occurred in in-person programming, and (2) learn about pandemic-specific, evidence-informed mental health knowledge. Our unique approach highlighted what dogs can teach humans about health through their own care and daily activities. From April to June 2020, we developed a website, created 28 Facebook livestreams and 60 pre-recorded videos which featured therapy dogs and handlers, and cross-promoted on various social media platforms. Over three months, first a combined process-outcome evaluation helped us determine whether our activities contributed to the program’s goals. A subsequent needs assessment allowed us to elicit participant preferences for the program moving forward. This commentary reflects on these findings and our teams’ collective experiences to share our key lessons learned related to program personnel needs, therapy dog handler training and support requirements, and online programming prerequisites. This combined understanding is informing our current activities with the virtual program and should be of interest to other therapy dog programs transitioning online.

## 1. Introduction

The PAWS Your Stress Therapy Dog program is a partnership between the University of Saskatchewan (USask) and St. John Ambulance (StJA), an international humanitarian organization [1]. The program is also offered in partnership with the USask community (i.e., libraries, the President’s office, Peer Health, Western College of Veterinary Medicine). Typically, therapy dogs visit the campus in-person during 1-h sessions every two weeks throughout the academic year. Students, faculty and staff attend to access the love/comfort (understood as “having reciprocal love for the dogs and gaining positive feelings from visiting with them” (pp. 332–333)), and support (“de-stressing and relaxing by interacting with the dogs” (p. 333) that therapy dogs have been recognized to provide [2,3,4,5]. Visiting therapy dogs are one type of an animal assisted intervention (AAI). The program was temporarily suspended in March 2020 when USask closed access to the campus due to the COVID-19 (i.e., an infectious disease caused by the coronavirus, SARS-CoV-2, a respiratory pathogen) pandemic [6].

Despite our team’s lack of preparation for these changes, we quickly transitioned the PAWS Your Stress Therapy Dog program into a novel virtual format with one-time funding from the Saskatchewan Health Research Foundation Research Connections COVID-19 fund. We recognized within a month of the start of the pandemic that USask students, staff, and faculty may require mental health supports, particularly with the challenges of isolation and loneliness because of various federally and provincially enacted physical distancing measures. At the same time, the pandemic occurred in an era when social media is a key source of knowledge exchange. Our unique online format was designed for the USask community, and all Saskatchewanians, to virtually connect with the therapy dogs and acquire evidence-informed information about pandemic-specific mental health self-care tips. Unique to our approach of knowledge dissemination was highlighting what dogs can potentially teach humans about health through their own care and daily activities. For example, our team leader and therapy dog handler, Colleen Dell, live-streamed a video of a therapy dog, Anna-Belle, enjoying skateboarding, and linked that to doing outdoor activities and hobbies which are important for mental health.

The purpose of this commentary is to highlight how our team transitioned the USask PAWS Your Stress Therapy Dog program from an in-person to a unique online format and the key lessons our team learned. These key lessons are in the areas of program personnel needs (i.e., staff time required, securing handler participation), handler training and support requirements (i.e., technology challenges, online expectations for therapy dogs, development of mental health tips and a resource guide) and online programming prerequisites (i.e., re-navigate known platforms, use YouTube as a learning guide, manage social media, analytics difficulties, using “traditional” media such as broadcast television). These lessons are informing our current and planned activities for the online program. It is anticipated that therapy dogs will not be back on the USask campus until the province fully reopens, which most likely will not occur until a COVID-19 vaccine is widely available. This aligns with the Centers for Disease Control and Prevention’s COVID-19-specific *Guidance for Handlers of Service and Therapy Animals* [7].

### PAWS Your Stress In-Person Program

With approval from the USask Animal Research Ethics Board (#20130115), the in-person PAWS Your Stress Therapy Dog program was initiated on the USask campus in 2015 by the office of the Centennial Enhancement Chair in One Health and Wellness and in partnership with StJA. The program also partners closely with USask Peer Health to make mental health resources available to student attendees. The program is an animal assisted activity (AAA), which is understood to be informal, typically delivered by a volunteer in partnership with an animal, and aims to enhance participants’ quality of life [8]. As shared, the goal of the visiting therapy dog teams at USask is to offer program participants feelings of love/comfort and support [9]. A therapy dog team is made up of a volunteer human handler and their companion dog, with both having undergone suitability screening (e.g., dog is non-aggressive) and passing the national therapy dog/handler test from StJA (e.g., dog indicates it enjoys visiting, human is personable). A typical in-person visit involves students meeting with several therapy dog teams in a designated space on campus. During the academic year, these visits were typically scheduled for 1 h on a bi-weekly basis, and averaged 150 attendees per hour with three visiting therapy dog teams. A typical in-person session involves students petting the therapy dog in a circle of approximately 4–5 other students and the handler. Literature supports the impact of petting an animal in AAAs for beneficial physiological changes, including decreased respiration rate, decrease in stress hormones (e.g., cortisol) and increase in feel good hormones (e.g., oxytocin) [10,11,12]. Conversation commonly focuses on school and pet related topics. The program has grown substantially since its beginning, with visits being offered on campus to students as well as faculty and staff, and extending to one-on-one settings for the purpose of offering support for individuals in a counselling setting. A brief promotional video and description of the PAWS Your Stress program is available (https://www.youtube.com/watch?v=BlDP5FkuudY accessed on 22 March 2021) [13].

The PAWS Your Stress Therapy Dog program is involved in ongoing research and evaluation. A 2015 evaluation of the immediate outcomes of the in-person program found that the program goals of offering students love/comfort and support were met. As shared, love/comfort was understood as “having reciprocal love for the dogs and gaining positive feelings from visiting with them” [5] (pp. 332–333) and support was understood as “de-stressing and relaxing by interacting with the dogs” [5] (p. 333). We address the concepts of love/comfort together because although the original goal of the StJA therapy dog program was to offer participants specifically with feelings of love and support, we have experienced some participant discomfort and confusion early on in the setup of the program with the term love. In response, love is often presented alongside the term comfort for clarity. A three-month follow-up with the sample of USask participants indicated that they recalled the visit with positive and happy feelings. A 2017 in-depth, qualitative examination of student experiences with the program likewise found that the attendees felt loved/comforted and supported. Students specifically reported that the program provided opportunities to be in-the-moment (e.g., forget their daily worries), facilitate social connections (e.g., with the dogs, handlers and other attendees), and complemented their existing healthy coping skills (e.g., taking a break) [14]. Such benefits of in-person visits have also been identified in other similar campus therapy dog programs (e.g., [15,16,17,18]). Less research details faculty and staff therapy dog visiting outcomes, but that which does exist indicates similar although anecdotal evidence where attendees’ emotional wellbeing benefits [19,20].

## 2. Online Transition

Several factors influenced our team’s decision to offer the PAWS Your Stress Therapy Dog program in a virtual environment. To start, we recognized the stress the pandemic was having on individuals’ lives. Globally, people were also reporting an increased sense of isolation and loneliness as a consequence of physical distancing and lock down measures [21,22]. At the same time, we learned that the Carleton University [23] therapy dog program was broadcasting live on Instagram. This aligned with the adaptation of university classes and other services to an online format at USask and elsewhere. We also observed a social media content trend focussed on the comfort that companion animals were providing to humans during these unprecedented times, including increased adoptions from animal shelters [24,25]. We knew as well that our handler teams were missing the opportunities to contribute to the community through their volunteer efforts, and that putting the planned expansion of the PAWS Your Stress program on hold was a disappointment to segments of the USask community. Finally, we considered that our entire community was experiencing the pandemic in an era of online communication.

Fortunately, the lead member of our team, Colleen Dell, also had some online therapy dog visiting experiences to draw upon. She has been managing an active therapy dog Facebook community of approximately 2000 followers for the past 7 years (@AnnaBelleSubiesAdventures). She is also the co-lead with Dr. Darlene Chalmers on a prison Canine Assisted Learning program, started in 2016, where bi-monthly video conference visits with participants take place following the in-person component of the program. Facilitating a virtual connection with therapy dogs was not entirely new to the team.

In the midst of considering how to offer our therapy dog program online, Pet Partners released a new initiative, titled Animal Related Engagement (ARE). ARE is defined as “[a]ny engagement opportunity that allows participants the benefits of the human–animal bond by encouraging the remembrance of feelings that are commonly associated with interaction with an animal” [26]. With our online program, we envisioned re-connecting with individuals who had visited with the therapy dogs in-person as well as with individuals who have had a pet in the past. We also envisioned the online program making new memories for individuals tuning in and positively affecting their wellbeing. 

Our team is knowledgeable about the importance of memories in therapy dog programming through our own experiences with sharing therapy dog photos and trading cards with program participants for several years and in various environments and related research. For example, our team was involved in a 2018 study of the effect of therapy dogs on the wellbeing of older Veterans living in a long-term care residence. It was found that the therapy dog visits had “a positive influence on memory recollection and reminiscence among [V]eterans” [27] (p. 83). Further, our project lead co-authored the 2019 Animal Memories magazine publication, which featured animal memories by inmates in Canadian correctional institutions (https://www.flipsnack.com/harperg/animal-memories-fcfexaf97/full-view.html accessed on 22 March 2021) and used virtual technology to connect with prison sites across the country to share these memories [28]. 

### 2.1. PAWS Your Stress Online Program

Our team began transitioning its in-person program to an online format by developing a website (www.therapydogs.ca accessed on 22 March 2021) to serve as a central hub to collate our varied online activities, posting social media content, as well as sharing information about scheduled activities. We also expanded our existing and adopted new social media platforms, including Facebook and Facebook Live (@PawsYourStress), Instagram (PawsYourStress), Twitter (PawsStress), Flipgrid (https://flipgrid.com/therapydogs accessed on 22 March 2021) and YouTube (https://www.youtube.com/channel/UCktTwbHipNjEReDp9IahWhQ/videos accessed on 22 March 2021). We intentionally chose online platforms that could store our program content to ensure we were participant centered; that is, meeting individuals where they are at, so they can view the content at their convenience. At all times, participants were encouraged to post questions and offer feedback. For this reason, Facebook and Instagram were specifically focussed on during the transition as we had an existing account with some followers to build upon. Facebook and Instagram were also experienced as popular social media on the university campus by our team and our program partner, Peer Health.

The recordings were cross-promoted on popular Facebook pages, including St. John Ambulance SK (7.2k followers), USask Peer Health (1k followers), and Colleen Dell’s Anna-Belle and Subie’s Adventures (2k followers) with hashtags (e.g., #OnlineTherapyDogs). Several of our therapy dog handlers cross-promoted our program on their Instagram, Twitter and Facebook accounts, and all team members and participants were encouraged to share the posts on their personal social media platforms. We also promoted the therapy dogs via internal USask communications. Efforts were also dedicated to advertising the visits to Saskatchewanians and other university therapy dog programs in Canada, including using paid Facebook advertisements, emails to social networks, press releases, and news articles (e.g., Saskatoon Star Phoenix, CTV Saskatoon) and which were picked up by various groups (e.g., Saskatchewan Health Authority, Yahoo News).

Our live and pre-recorded videos featured the therapy dogs and handlers engaging in various activities they regularly engaged in and linked these activities to an evidence-based pandemic-specific mental health self-care tip, such as going for a walk. Additional activities that the dogs and handlers engaged in ranged from a healthy food taste test between a therapy dog in training and a therapy dog, training with a Canadian Kennel Club recognized trainer, grooming for a dog show, taking photos outdoors, and a dog skin care tutorial. Rarely, if ever, were the dogs asked to engage in activities they were not already familiar with.

The Facebook live visits were on average 15 min, mirroring the typical length of a participant’s in-person therapy dog visit. The pre-recorded videos varied in length, with some as short as 2 min and were more of a greeting style. The Instagram videos were typically a maximum of 1 min, adhering to the site format, unless we uploaded to the specific Instagram TV feature. Several special videos were compiled of various canine companion animals of members of our team to advertise the program. There were also regular social media posts with photos of the therapy dogs staying healthy and active, and again linked with a positive and encouraging mental health message. It is important to note that in the period of time before we began the recordings, our team posted regularly on social media with updates from the therapy dog handler teams. These “check-ins” took the form of pictures and stories regarding what the dogs were up to while unable to visit in person. This was met with positive follower engagement. We also posted pre-recorded children’s stories read to therapy dogs by their handlers, in partnership with Scholastic Canada and authors Nicole Petroski and Jane Smith.

### 2.2. PAWS Your Stress Program Output, Evaluation and Reflection

As a university-based program, our team is well aware of the need and equipped to design and undertake planning and evaluation of the PAWS Your Stress Therapy Dog program, including during the rapid online transition. Given the novelty of the online program and urgency with which the pandemic was experienced, it was important to offer the programming swiftly, as well as undertake evaluation, and document our team’s reflections about key lessons learned.

Our PAWS Your Stress program output between 22 April and 31 July 2020 included:

- 39 pandemic-specific mental health videos;
- 10 storybook reading videos (English or French);
- 28 Facebook Live videos and 11 pre-recorded videos containing pandemic-specific mental health self-care tips;
- One podcast episode with Be Well at USask (aired 22 May 2020);
- One infographic co-designed and promoted with the Canadian Centre on Substance Use and Addiction and the Mental Health Commission of Canada, titled Coping with Stress, Anxiety, and Substance Use During COVID-19: How Animals Can Help.

There were nine additional book readings and 23 videos created between these dates, but they did not include pandemic-specific mental health tips. As such, they are not reflected on in this commentary.

A combined process-outcome evaluation followed by a needs assessment was undertaken over a three-month period to determine whether our team’s activities contributed to achieving the program’s goals. That is, we wanted to know whether program participants: (1) experienced feelings of love, comfort, and support; and (2) learned about pandemic-specific mental health self-care knowledge. Contrary to the program’s history, we separated the terms love and comfort for this evaluation to respond to the unique on-line nature of the program and increased awareness of love being a therapy dog program goal since it was incepted in 2015. Unfortunately, due to time constraints based on our COVID-specific funding, we were unable to compare multiple time points to determine participant change. As such, we only assessed perceptions/opinions at a single time point. Quantitative and qualitative data were collected from the target population, USask students and community, as well as participating Saskatchewanians, with two online questionnaires (2 and 4 months after the inception of the online program; questionnaires available 8 to 19 June 2020 and 10 to 27 July 2020, respectively). Given the online and public nature of the program, it was not feasible to exclude non-USask community members from participating, although their participation was determined to be limited. We recognize that our on-line sampling approach was not ideal, but it is what we had access to doing at the time. Human Research Ethics Board approval was not required given the evaluative focus of our work.

The first process-outcome evaluation questionnaire was designed to assess participant opinion and experiences with the PAWS Your Stress online program. The questionnaire was accessible via Facebook, Instagram posts/stories and through the therapydogs.ca home webpage, and a total of 94 participants completed it, nearing our anticipated goal of 100. Most participants were registered students (26%), 18–25 years old (23%), self-identified as a woman (92%), resided in Saskatoon, Saskatchewan (65%), and currently had a pet (88%). In brief, we found the following: Related to Goal 1, participants were asked using a 5-point Likert-type scale (1 = strongly disagree to 5 = strongly agree) whether they felt comforted, loved by, connected to, and supported by the therapy dogs online. We decided to include the concept of connection given the growing emergence of it in the AAI field (e.g., informed by growing attention to the human–animal bond) and its possible linkage to memories. The majority of participants strongly or somewhat agreed that they felt comforted by (90%), loved by (63%), connected to (86%), and/or supported by (83%) the therapy dogs. Participants were also asked using a 5-point Likert-type scale (1 = A lot less to 5 = A lot more), compared to other sources of connection, love, comfort, and support in their lives, how much did they experience these feelings generally from watching the therapy dogs online. Most participants reported that they experienced these feelings a little bit more (39%) or about the same (30%) from watching the therapy dogs online (“Human–animal bonds are there whether online or in-person”—participant). In order to feel more connection, love, comfort, and/or support from the therapy dogs online, participants suggested providing more information about the dogs (“It would be nice to know their birthdays and favourite toys”—participant) and having the dogs engage more and in different activities (“Learn tricks as suggested by viewers”—participant). Related to Goal 2, most participants (68%) reported “yes” to learning about pandemic-specific mental health self-care tips from the handlers and therapy dogs online (Yes, No, Unsure response options). Using a 5-point Likert-type scale (1 = strongly disagree to 5 = strongly agree), participants were asked whether they were more aware about evidence-informed, pandemic-specific mental health self-care knowledge after watching the online videos and whether they were using the tips they learned from the online videos. The majority of participants strongly or somewhat agreed that they were more aware about (83%) and were using (85%) the pandemic-specific mental health self-care tips from the therapy dog online videos. We also asked participants what made the pandemic-specific mental health self-care tips easy for them to apply. Participants indicated that the mental health tips were easy for them to apply because they felt inspired by the video content (“Watching nice landscapes and houses encouraged me to walk more around the neighbourhood and other areas”—participant), and also identified them as good reminders (“They were reminders to take breaks and take care of yourself”—participant). The tips were recognized as simple, clearly explained, affordable, and attainable (“They were not complex. Rather, they were put in simple, plain words and ideas”—participant). While many individuals noted that they needed to figure out for themselves how to increase their motivation and the likelihood that they would apply the self-care tips (“I think I was doing what I could already. The therapy dogs were a break in my day that often made me smile”—participant), some suggested that the tips needed to be communicated differently (e.g., more enthusiasm, tailored to specific populations, etc.) and that even more or different tips could be offered (“Reinforcement of their importance. Perhaps in subsequent videos, getting the same dogs to reinforce how those specific tips help them or how they are already practicing or applying the tips”—participant).

The second needs assessment questionnaire assessed participant needs and preferences regarding program implementation to help inform future programming. The questionnaire was again advertised through Facebook, Instagram, and the therapydogs.ca home page, and had a total response of 372 participants. We anticipate the increased response compared to the first survey was due to broader awareness and engagement with www.therapydogs.ca (accessed on 22 March 2021). Most participants were full-time students (50%), between 21 and 25 years old (26%), self-identified as a woman (82%), and currently had a pet (72%). Findings from this questionnaire also supported the two main outcomes we sought to measure. In line with Goal 1, participants were asked using a checklist of possible response options (e.g., stress reduction, loneliness, learn about the therapy dogs, etc.) why they want to visit with the therapy dogs online. Participants primarily visited the therapy dogs online to reduce stress (48%), to learn about the therapy dogs’ lives (37%), and because they missed the therapy dogs (29%) (“Just seeing the dogs do their thing brings me joy”—participant). There was no specific data collected in our second questionnaire pertaining to our Second Goal about increasing pandemic-specific knowledge, however, 16% (58) of respondents indicated that they wanted to visit the therapy dogs online “to learn how to stay healthy during the COVID-19 pandemic” [29] (p. 7).

Reflecting upon these evaluation outcomes and our team’s unique 14-week implementation and delivery of the PAWS Your Stress Therapy Dog program online, prompted us to reflect on the key lessons learned. Consequently, we are motivated to continue to offer the program virtually, as well as consider how it could be improved to meet the needs of our target USask population. The key lessons we learned are in the areas of program personnel needs, handler training and support requirements, and online programming prerequisites. This combined understanding is informing our current activities with the virtual program and should be of interest to other therapy dog programs transitioning online.

## 3. Key Lessons Learned

Looking back, the transition from an in-person program to an online program in the face of a pandemic was a trying time. Our team questioned at the start if it was even possible or worthwhile. We quickly learned through experience and evaluation that it was and that the program could still have a positive impact in the lives of individuals who were accessing our program. We have learned several key lessons along the way (Table S1). These are important to document because there may be value to revise and/or expand our online therapy dog program during COVID-19, possibly continue certain components of it post-COVID-19, and inform others who are considering it or undertaking it at present.

### 3.1. Program Personnel Needs

The first lesson our team learned was specific to our human staffing requirements. Our multi-disciplinary team is comprised of a USask Sociology faculty member, two post-doctoral fellows, a Masters level practicum placement student, and three part-time research assistants. We work cumulatively in what would amount to 1.5 full-time positions on the PAWS Your Stress program. This is considerably more in comparison to the in-person program, which required a 0.5 time staff position. As a group, we also spent extensive time on Zoom and email initially brainstorming ideas amongst our team members and with our partners, thinking through the benefits and drawbacks of each idea (especially given that there was a very limited online therapy dog pool of knowledge to draw from), and putting it all into action in the program. While the team lead spent considerable time upfront, this decreased as the program established itself.

There was likewise participation of the therapy dog teams to consider. The PAWS Your Stress program typically had 35 handler and therapy dog teams to draw from to visit campus. With the transition to an online format, we learned that the majority of handlers were reluctant to the change to an online format, some were hesitant, and some were very excited for the new program. The primary reason for handlers’ resistance was the permanence of online platforms. In retrospect, we recognize that Instagram may have been a more compatible choice for some as the platform does not store live video content online after 24 h. Additionally, without all of the regular therapy dogs participating, some students were unable to continue connections with dogs that had visited in-person. However, we chose the platforms we did so individuals could access them when they wanted to. In the end, we had a total of 17 handlers participating in the Facebook Live events, 18 in posting photos, and 24 in recorded greetings and story book readings. For the participating handlers, their openness to technology and desire to connect with students and the broader community through their therapy dog drove their decision to participate. The number of handler/dog teams ended up being enough to post live videos 2–5 times per week at the beginning of the project, and this reduced as we transitioned from spring to summer months, which is typical for the program. The amount of content produced at the beginning of the project was not limited by handler engagement.

### 3.2. Therapy Dog Handler Training and Support Requirements

We recognized early on that for handlers to participate comfortably and effectively online they required training with the technology we were using. When we started the online platforms, two of our research assistants connected individually and over Zoom with the handlers to do introductory training on how to use Facebook Live and Zoom as well as how to install the necessary applications on their device and establish user accounts. This was generally done by a staff member and 2–3 therapy dog teams at a time online or over the phone. Sessions generally lasted 1 h and handler confidence increased as they engaged with the technology. A research assistant was also available via text to assist with technological difficulties whenever a handler was livestreaming on Facebook Live. Difficulties that arose ranged from holding the camera sideways through to poor sound quality in a windy environment. We also recognized that access to online connections varied for participants. The ease and quality of online work can depend on the device being used (e.g., phone vs. tablet), and access to technology (e.g., Wi-Fi not available during outdoor activities, data plan costs, internet speed).

Handlers also required support with navigating their therapy dogs’ role during online visits. Consulting periodically with an established dog trainer to include their expertise, we shared with the handlers that while the dogs were not able to recognize the technology or that there is a camera on them, they do recognize voices or audio output from the devices (although that was not relevant to our recordings). We suggested that the handlers establish an enjoyable routine with and for their dog while being in front of the camera. Depending on the handler and dog, this may involve, for example, cuddles, treats and/or clicker training. This was explained as an indirect connection with the audience by the dog, and more directly by the handler; this is a role reversal from in-person visits. Many of the handlers reported that the dogs began to recognize and appreciate the routine during the online visits. Animal welfare is the cornerstone of the PAWS Your Stress program, and we navigated this carefully with the on-line transition, encouraging handlers to be involved in activities with their dogs that they typically do. This was relatively easy to accomplish with our initial COVID-19 focus and messaging being specific to the dogs engaging in daily activities that they enjoyed for their own care. This includes recognition of the reciprocal relationship the dog and handler posses. Consideration of the dogs’ perspectives is inline with attention to animal welfare, ethics and needs in the new e-resource Alternatives to Traditional Animal Assisted Interventions: Expanding Our Toolkit (2020) [30].

Our team anticipated the need to collate evidence-informed mental health tips for the therapy dog handlers to relay. Doing so also ensured that the shared tips were clear, consistent and “on script.” We considered this to be important during the pandemic and did all we could to ensure supportive and evidence-informed messaging was being shared. Our team had access to and examined peer-reviewed and grey literature focused on pandemic-specific mental health tips from various health organizations. These included the World Health Organization [31], Public Health Agency of Canada [32], Psychiatric Times [33], the Centre for Addiction and Mental Health [34], and the Mental Health Commission of Canada [35]. Drawing on these resources and team member Colleen Dell’s experience facilitating online visiting in other contexts, we developed a handler handbook that included examples of mental health tips that are relevant to both dogs and humans, activities, and scripts for Facebook Live events and Zoom book readings, as well as how to use technology. The handbook also informed handlers of what is expected of them, and they could decide to opt out after reviewing it. At the same time, it eased the burden on the handlers to know these expectations and helped increase their confidence.

### 3.3. Online Programming Prerequisites

Our team chose to begin the online transition of the PAWS Your Stress program by engaging with platforms we had some collective experience with, as the stress of the pandemic on other aspects of our lives, jobs and education was not conducive to doing otherwise. We used GoDaddy to build the website because it does not require a website design background and is moderately priced. That said, developing website content that is easy to navigate is a skill that none of our team had expertise in and we could still benefit from. We used YouTube as a hub for all the videos we produced. The platform allows for multiple users and unlimited storage of videos, and videos in the production phase could be designated as private or “unpublished” for all team members to access without being seen by the general public. We also used Facebook, Twitter, Instagram, and Flipgrid because these were believed to be most popular amongst the USask community, and again, we were familiar with them to some extent. However, each platform had its drawbacks. For example, a downfall of using YouTube as our video hub was that it required significant time to convert the videos to store there. Additionally, Twitter had limited use by our audience and so was slowly phased out of use during the program, Instagram showed but did not store our live videos, handlers found Flipgrid challenging to use, and not all handlers were willing to acquire a Facebook account for principle-based or personal reasons.

Our team also used YouTube to search for videos to learn about the social media platforms we were using. This included learning how to cross-post to multiple platforms to save time and reach diverse audiences (e.g., between Facebook and Instagram). Using multiple platforms was important because it allowed for varied reach as well as diverse forms of audience engagement (e.g., comments, likes/reacts, shares). We also searched for the prime times to post content (e.g., Monday through Thursday early afternoons on Facebook, posting twice per week on YouTube). Additionally, we learned how to edit a recorded Facebook Live video before they were posted to our website (i.e., after a live event) to remove, for example, political content or the home address of a handler that was mistakenly shown. We quickly learned too that technology was constantly changing, including YouTube and other social media, and this was especially true during the pandemic as the platforms competed to secure users.

Our team learned that managing social media is time intensive. One example is trying to ensure consistency in the look, feel, and content of our messaging (i.e., branding), especially when multiple team members are involved in developing it. The hiring of a digital marketer would be extremely useful. We also rarely engaged with social media on the weekends or in the evenings. This could be revisited, given our survey finding that participants indicated they would like to visit the therapy dogs on weekday evenings, as well as the weekend [29]. We also recently looked into Hootsuite, a program that allows for the advanced scheduling of posts across platforms by multiple users [36]. We confirmed that engaging the handlers during the summer was challenging, as they departed on holidays, including during the pandemic. We knew handlers often took the summer off, and this was one reason the on-campus therapy dog program did not offer regular visits during the summer months. In order to foster more regular engagement, our team returned to establishing weekly scheduling of the handlers to do Facebook Live streams, as we had for in-person visits, aiming for two per week as well as three pre-recorded videos. Additionally, a last key lesson learned by our team is that a moderator is regularly required on the social media sites to ensure appropriate comments, etc. A moderator also helped pose questions from the online chat to the handlers during Facebook Live streams. We are fortunate that our program had little to no unfavorable sharing by attendees.

Our team also learned that interpreting social media “analytics” or “insights” is not straightforward and most often cannot be compared across platforms. From what we could determine, on all of the platforms that the PAWS your Stress program engaged with, we were able to increase subscribers and views over time. It is not clear from the available analytics, however, how long individuals engaged with each view (e.g., did they watch a full video). Due to the varied analytics across platforms and other factors, we were not able to determine which advertising techniques were most effective at gaining subscribers or views. We relied on data from Google Analytics for our website, which does not have analytics embedded in it from GoDaddy.

Analytics we found meaningful include the following. Across all the pandemic-specific videos, there were 587 comments from viewers over the three-month period. Types of comments included: questions/comments regarding general and therapy-specific dog training; questions/comments about the dog, including in-person time spent with the dogs and sharing special memories; compliments for the handler and expressions of gratitude for making the video; greetings for the therapy dog team; non-specific compliments and expressions of gratitude; and, comments about how the videos made viewers feel. Facebook Page likes increased from 199 on 13 April to 705 on 31 July 2020, with an average of 7 new page likes per day, and followers increased from 209 on 13 April to 789 on 31 July 2020. As another example, there was a total of 21 Flipgrid videos, 6 pandemic-specific, with 1183 views that translated into 33.9 h of total viewing. Our YouTube videos had an average of 130 views. It is important to mention though that most of our viewers were tuning in to videos specifically through Facebook Live or Instagram (after the fact), and so are not recorded on YouTube analytics. Our videos with the most views on YouTube included “Anna-Belle the therapy dog reads Birthday Party Pandemonium” (330 views), “Kisbey the therapy dog reads Three Easy Steps to Getting a Dog” (221 views), and “Murphy the therapy dog reads Murphy Mondays!”, which is a book written about him (148 views). We recognize that these analytics may not be impressive in comparison to other social media sites and activity but given the growth from the beginning to end points for this paper we are happy with what we achieved given that we are still in what is arguably the establishment phase of the online program and amidst a pandemic.

Our team also used more “traditional” media to market our program, including local news media and newsletters as well as information sharing technologies within our university (e.g., e-mail and e-lists). We tried to pay attention to the audience’s preferred formats (e.g., photos, videos, infographics) and culture. This included advertising techniques that used the USask channels focused on stresses experienced by this population, used language commonly applied across university communications, and referred to the in-person therapy dog program that would normally be available. For advertising efforts beyond the university environment, more general COVID-19-related stressors were acknowledged and evidence of the efficacy of therapy dogs was highlighted. Social media posts regularly used informal language and utilized humour to engage all of our audiences.

## 4. Conclusions and Future Directions

This commentary has documented highlights of our team’s transition from an in-person to an online therapy dog program, while also reflecting upon evaluation outcomes and multiple lessons learned throughout the transition and implementation of the virtual program. These are in the key areas of program personnel needs, therapy dog handler training and support requirements, and online programming prerequisites. It is clear to our team that individuals enjoy and want to connect with the therapy dogs online as physical distancing mandates remain in place. While there is no substitute for touching and interacting with the therapy dogs in-person, attendees reported to enjoy seeing the dogs, connecting with or maintaining connections with them, learning about them, and gain cognitive/emotional benefits (e.g., stress reduction). Many attendees have also reported feelings of comfort, love, connection, and support from the therapy dogs in our on-line evaluations, similar to in-person findings. These findings support the 2015 in-person program evaluation and the 2017 in-person PAWS Your Stress study that reported students identified the program as providing opportunities to be in-the-moment (e.g., forget their daily worries), facilitate social connections (e.g., with the dogs, handlers and other attendees), and complemented their existing healthy coping skills (e.g., taking a break).

Moving forward, we understand from our reflective experiences and evaluations that most participants of the PAWS Your Stress program would like to learn more about the dogs’ lives and therapy dog training in general. Some also suggested more audience participation, having less focus on the handlers with more focus on the dogs, and requested for the video and sound quality to be improved. They indicated that Instagram, Facebook, YouTube, and therapydogs.ca are the preferred platforms for accessing therapy dog videos and photos. Videos should be brief (between 1 and 5 min), and if online meetings are offered via Zoom, they should be a maximum of 20-min sessions. We also noted that Facebook boosts should be utilized as they greatly increased video views (though may not translate into followers), and that there is no need to include pandemic-specific mental health information in our videos as audiences were most interested in simply seeing and connecting with the therapy dogs.

In response, our team is undertaking the following next steps. First, we will revise our social media platform focus. This will include transitioning to non-COVID-19 specific Facebook Live streams and photo posts on Facebook and Instagram, while continuing to transition away from using Twitter and Flipgrid due to low response, and reducing the number of Live videos. We will also attempt to draw upon a social media expert to best target and offer the program, keep up with the rapidly changing technology, and participants’ online “overload.” Second, we will host Zoom sessions for the USask and broader Saskatchewan community. These will follow a conference call format with the Zoom Breakout Rooms feature, which allows for attendees to split off into a separate session from the main meeting and interact with the therapy dogs in a more intimate format [37]. They can then move between or enter different Breakout Rooms to visit with the other therapy dogs. Third, we will advertise the content made for this project with specific communities, starting with Saskatoon hospitals through specially developed life-size therapy dog cardboard cut out signage and posters directing patients to www.therapydogs.ca (accessed on 22 March 2021) while in-person visiting is prohibited. Throughout online delivery, we will continue with our process and outcome evaluation, through which we will pay particular attention to whether there is a downside to reconnecting with dogs that were once visited with in-person (e.g., worsened feelings of isolation by not being able to physically interact). Fourth, our team will begin to explore designing a project to directly compare on-line versus in-person therapy dog visiting for when in-person visiting resumes, noting the two programs varied significantly in their formats. We are particularly interested in the role of the handler, which has received limited attention generally within the therapy dog research realm. We are also interested in learning how participants experience love and comfort as distinct concepts, as well as their understanding and experience of connection (with both the therapy dogs and handlers) and a possible linkage with memories. Finally, we will continue our evaluation of the PAWS Your Stress program and for various demographics (e.g., accounting for gender, age, vulnerability), including assessing for participant cognitive and emotional changes by comparing multiple time points, which we were unable to do for this evaluation due to time and resource constraints. 

As shared, it is anticipated that therapy dogs will not be back on the USask campus until the province fully reopens, and this is not expected to be in the near future. We understand many therapy dog programs are in the same position, although some have started revisiting outside of our province of Saskatchewan. We offered our key lessons learned through uniquely transitioning the PAWS Your Stress program online and our plans for moving forward in the hope that they will be useful for other therapy dog programs, and possibly even AAI clinicians who have, or are considering, facilitating AAIs in virtual settings during this time when many people are experiencing increased isolation and stress, or beyond. In particular, we suggest that involving animals in online therapy sessions could positively impact therapeutic relationships between the clinician and client, given that existing research has demonstrated that animal involvement in therapeutic settings can facilitate the establishment of a stronger therapeutic alliance [38,39]. Research has also demonstrated the efficacy of online therapy [40,41,42], which is emerging as an additional support to conventional face-to-face counselling especially, and most recently, gaining traction due to the COVID-19 pandemic. Mental health clinicians can apply these lessons learned when preparing to facilitate online therapy involving their animal or, preferably the client’s animal in recognition of the benefits of the human–animal bond. This approach, whether online or in-person, also requires that a clinician possesses an animal-informed understanding of the multitude of resources offered by the inclusion of an animal in the therapeutic context [43]. We recommend clinicians considering facilitating AAIs virtually consult the e-book Alternatives to Traditional Animal Assisted Interventions: Expanding Our Toolkit, and continue to be attentive to animal welfare concerns when facilitating AAIs in virtual settings and, in the future, in-person [30]. Specifically communicated in the above mentioned toolkit is that “having knowledge of your animal’s behavior at a species, breed, and individual level is crucial to maintaining [their] safety and welfare” (8:2020). Additional important considerations include, for example, if and how the dog is freely expressing themselves during the online session and the reciprocal nature of their relationship with the handler. Our team is currently working on a critical reflection of dog specific ethical considerations in the program’s transition to accompany this human-focussed commentary. 

While we transitioned the PAWS Your Stress program online due to necessity, this novel experience has highlighted the potential for online engagement with AAIs beyond the pandemic: to reach individuals living in rural and remote communities, individuals who are immunocompromised, and individuals whose allergies interfere with them being in the same room with certain animals. This is likely something our team will continue to explore and engage in. The lessons learned will have implications as we move forward dealing with the pandemic and afterward, as the greater society gains increased experience and comfort with the online world. 

## Data Availability

Data can be accessed through www.therapydogs.ca (accessed on 22 March 2021).

## Supplementary Materials

The following are available online at https://www.mdpi.com/2076-2615/11/3/914/s1, Table S1: Key Lessons Learned for Transitioning a Therapy Dog Program Online.
