# Supplementary material for: A Commentary about Lessons Learned: Transitioning a Therapy Dog Program Online during the COVID-19 Pandemic
Source: Animals (Basel). 2021 Mar 23;11(3):914. doi: 10.3390/ani11030914 (PMC8004773; doi:10.3390/ani11030914)
Supplement: Supplementary file 1 [file animals-11-00914-s001.pdf]

Table S1. Key Lessons Learned for Transitioning a Therapy Dog Program Online.

1. Program personnel needs	There are increased human staffing requirements. Our transition and ongoing offering of the online program equals a 1.5 full–time staff position, compared to a .5 time staff position for in-person programming.
	Handler participation is not guaranteed for online programming. Visiting online and with the necessary technology will be new for some handlers, and not all will want to participate.
2. Therapy dog handler training and support requirements	Anticipate technology challenges. As shared, the technology required for visiting online will be new for some handlers and so they will require training and support. Anticipate complications due to limited data plans, Wi-Fi coverage, device quality, etc.
	There are new expectations for therapy dogs when visiting online. An online visit is not comparable to an in-person visit for the therapy dogs. Handlers need to establish an enjoyable routine with and for the dog while in front of the camera. The handler has a much more facilitative role in on-line visiting.
	The development of mental health tips and a resource guide for the handlers is suggested. Mental health during COVID-19 is not an area of expertise for the majority of handlers, and so a guide will help ensure that the knowledge shared is clear, consistent, evidence-based and “on script.” This will ease the burden on the handlers as well.
3. Online programming prerequisites	When choosing an online platform to get started, start with what platforms are already known and introduce new ones that are easy to access and use.
	Use YouTube as a learning guide. YouTube often provides simple instruction for using social media platforms, which can be complex. Platforms are changing quickly during COVID-19 and it YouTube videos and channels can help you keep up-to-date.
	Managing social media is time intensive. This can range from ensuring consistent messaging amongst multiple team members, through to requiring a moderator to monitor the chat function during a live online event.
	Social media analytics can be difficult to access, compare and interpret.
	Traditional media should be continued to market the online program. This may include broadcast television, such as local news media and newsletters as well as information sharing technologies within your university.
